# Asthma and Keratoconus: A Vision at Risk and the Urgency of Early Screening

**DOI:** 10.12669/pjms.41.11.12696

**Published:** 2025-11

**Authors:** Zainab Junaid, Nisha Babar

**Affiliations:** 1 Zainab Junaid, Dow University of Health Sciences, Karachi, Pakistan; 2 Nisha Babar, Dow University of Health Sciences, Karachi, Pakistan

Keratoconus or KC is a progressive eye disease where the cornea thins and bulges outward, usually starting in adolescence or young adulthood and often causing significant vision loss.^1^ Although we know genetics and environmental triggers like frequent eye rubbing and family history play a role, the specific biological mechanisms of KC progressions are still unclear.^1^ Epidemiological studies have repeatedly observed links between KC and allergic conditions, including asthma. One large analysis by Hashemi et al. calculated that people with asthma have nearly double the risk of developing KC, with an odds ratio around 1.94.^2^ However, researchers suggested that this association might be complicated by factors like eye rubbing, which is more common in people with allergies.^2^ More recently, a powerful genetic study by Yang et al. using two-sample Mendelian Randomization or MR (a method leveraging genetic variants as proxies for disease exposure) shed clearer light on the relationship.^3^ Analyzing genetic data (GWAS) from over a million European-ancestry individuals, they found that allergic asthma increased the risk of KC (odds ratio = 2.18).^3^ No causative relationship was established for other allergic illnesses such as atopic dermatitis, urticaria, or allergic conjunctivitis, revealing a unique link between allergic asthma and KC.^3^ Yang et al. identified allergic asthma as a causal risk factor for KC but did not investigate underlying mechanisms.^3^ However, earlier research has linked inflammation to KC. For instance, Lema and Durán documented that patients with keratoconus had elevated levels of inflammatory cytokines IL-6 and TNF-α, suggesting inflammation is part of the disease process.^4^ We know these cytokines drive inflammation in asthma too. So, could there be a common link between the two diseases? To answer that, we’d need studies directly comparing cytokine profiles in KC and asthma patients simultaneously. The study’s conclusions are primarily drawn from European populations data.^3^ Confirming these findings in other diverse ethnic groups will be critical for broader applicability.^3^ Clinically, these findings emphasize the necessity of greater clinical vigilance for ocular symptoms in asthma patients, since diagnosing it early allows for prompt interventions such as corneal collagen cross-linking^1^ to reduce or prevent keratoconus progression. There is also an urgent need to research the genetic and inflammatory processes that link the two illnesses, as this will inform future targeted therapies such as immunomodulatory medicines. Moreover, collaboration between respiratory and eye care specialists must be strengthened as delayed detection of this link might result in irreparable vision loss.

## References

[1] Gomes JA, Tan D, Rapuano CJ, Belin MW, Ambrósio R, Guell JL (2015). Global consensus on keratoconus and ectatic diseases. Cornea.

[2] Hashemi H, Heydarian S, Hooshmand E, Saatchi M, Yekta A, Aghamirsalim M (2020). The Prevalence and Risk Factors for Keratoconus:A Systematic Review and Meta-Analysis. Cornea.

[3] Yang X, Yu X, Gao S, Wei P, Han G (2025). Causal Relationship Between Common Allergic Diseases and Keratoconus:A Two-Sample Mendelian Randomization Study. Transl Vis Sci Technol.

[4] Lema I, Durán JA (2005). Inflammatory molecules in the tears of patients with keratoconus. Ophthalmology.

